# Autoantibody to GNAS in Early Detection of Hepatocellular Carcinoma: A Large-Scale Sample Study Combined with Verification in Serial Sera from HCC Patients

**DOI:** 10.3390/biomedicines10010097

**Published:** 2022-01-04

**Authors:** Xiao Wang, Keyan Wang, Cuipeng Qiu, Bofei Wang, Xiaojun Zhang, Yangcheng Ma, Liping Dai, Jian-Ying Zhang

**Affiliations:** 1 Department of Biological Sciences & NIH-Sponsored Border Biomedical Research Center, The University of Texas at El Paso, El Paso, TX 79968, USA; xwang6@utep.edu (X.W.); cqiu3@utep.edu (C.Q.); bwang2@miners.utep.edu (B.W.); xzhang5@miners.utep.edu (X.Z.); yma3@miners.utep.edu (Y.M.); 2 Henan Institute of Medical and Pharmaceutical Sciences, Zhengzhou University, Zhengzhou 450052, China; keyanwang@zzu.edu.cn

**Keywords:** hepatocellular carcinoma, tumor-associated antigens, autoantibodies to tumor-associated antigens, biomarker, early detection

## Abstract

The aim of this study was to explore the value of autoantibody to GNAS in the early detection of hepatocellular carcinoma (HCC). In a large-scale sample set of 912 participants (228 cases in each of HCC, liver cirrhosis (LC), chronic hepatitis B (CHB), and normal controls (NCs) groups), autoantibody to GNAS was detected with a positive result in 47.8% of HCC patients, which was significantly higher than that in patients with LC (35.1%), CHB (19.7%), and NCs (19.7%). Further analysis showed that the frequency of autoantibody to GNAS started increasing in compensated cirrhosis patients (37.0%) with a jump in decompensated cirrhosis patients (53.2%) and reached a peak in early HCC patients (62.4%). The increasing autoantibody response to GNAS in patients at different stages was closely associated with the progression of chronic liver lesions. The result from 44 human serial sera demonstrated that 5 of 11 (45.5%) HCC patients had elevated autoantibody to GNAS before and/or at diagnosis of HCC. Moreover, 46.1% and 62.4% of high positive rates in alpha-fetoprotein (AFP) negative and early-stage HCC patients can supplement AFP in early detection of HCC. These findings suggest that autoantibody to GNAS could be used as a potential biomarker for the early detection of HCC.

## 1. Introduction

Hepatocellular carcinoma (HCC) is one of the most common cancers worldwide and is the third leading cause of cancer-related mortality [1]. Due to the lack of sensitive and reliable diagnostic methods, more than 60% of early-stage HCC patients could not be diagnosed [2]. The current diagnosis of HCC mainly relies on imaging examinations and serological markers. However, the tumor size in early-stage HCC patients is too small to be detected by imaging. Alpha-fetoprotein (AFP) is commonly used for the clinical diagnosis of HCC, but it has a limitation of lower sensitivity for the detection of early-stage HCC and small HCC. Moreover, many at-risk patients with chronic liver diseases also have an elevated level of AFP [3,4]. Therefore, it is paramount to explore more novel effective serological markers to supplement the roles of imaging and AFP for the early detection of HCC.

Autoantibodies against tumor-associated antigens (TAAs), appearing in patients sera years before clinical symptoms of cancer, can be used as biomarkers for the detection of cancer, as confirmed by many previous studies [5,6,7,8,9]. The changes of TAAs in quality and quantity, including mutation, overexpression or aberrant expression, post-translational modification, etc., can be recognized as heterologous antigens by the immune system to elicit a humoral immune response for producing corresponding autoantibodies [10,11,12]. Autoantibody to TAAs (TAAbs) is more stable and longer-lasting than other potential biomarkers, even TAAs [13,14]. Multiple autoantibody-based potential biomarkers for the detection of HCC were discovered in our previous studies [12,15]. In our recent study [16], more novel biomarkers were identified with an autoantibody to GNAS showing good performance in HCC detection.

Guanine nucleotide-binding protein Gs subunit alpha (GNAS) belongs to the G protein family. Heterotrimeric G proteins consist of alpha (a), beta (b), and gamma (r) subunits [17]. G proteins act as molecular switches inside cells, and function to relay transmitting signals from cell surface receptors to intracellular effectors. Most studies about G proteins have been focused on alpha subunits which have aroused more attention and research due to their relationship with cancers [18]. GNAS, one of the alpha-subunits, is a proto-oncogene originally described in pituitary adenomas [19,20]. A *GNAS* mutation, any change in the genetic sequence of GNAS, has been identified in a number of neoplasms including those of the lung, appendix, colon, pancreas, and kidney [21,22,23,24,25]. Studies on GNAS protein expression demonstrated that the high expression of the GNAS protein enhanced breast cancer cell proliferation and migration, as well as HCC cell growth and invasion [26,27]. Most studies on *GNAS* in cancers were performed at the gene level, and a few studies on GNAS were carried out at the protein level; however, none of them, except our recent study, are related to GNAS autoantibody in sera from patients with HCC [21,22,23,24,25,26,27]. Recently, we screened out 11 potential TAAbs from HCC patients by a focused protein microarray and autoantibody to GNAS was one of them [16]. However, in the study, the data analysis for individual autoantibody was not stratified and there was no clinical follow-up evaluation. The objective of the current study is to detect the levels and changes of autoantibody to GNAS in patients at different stages during the formation of HCC combined with verification in serial sera from patients with chronic liver disease-transformed HCC to explore its diagnostic value for the early detection of HCC.

## 2. Materials and Methods

### 2.1. Human Serum Samples

A total of 1149 human serum samples were included in this study. All serum samples except HCC successive sera were from the serum bank of the Tumor Epidemiology Laboratory of Zhengzhou University (Henan, China). All HCC patients in the study were diagnosed according to criteria established in 2017 in China [28]. The staging of HCC was defined based on the eighth edition of the American Joint Committee on Cancer (AJCC) Cancer Staging Manual [29] and the Barcelona Clinic Liver Cancer (BCLC) staging [30]. HCC patients at stage I–II in TNM staging or at stage 0-A-B in BCLC staging were defined as early-stage HCC patients, while HCC patients at stage III-IV in TNM staging or at stage C-D in BCLC staging were classified as late-stage HCC patients. Ninety-six sera from patients with HCC and 49 sera from normal controls (NCs) were collected for testing in a focused protein microarray. The validation set consisted of 228 patients with HCC, 228 patients with liver cirrhosis (LC), 228 patients with chronic hepatitis B (CHB), and 228 normal controls (NCs). The participants among the four groups were matched in age and gender except for CHB patients in age. All NC individuals had no history of liver diseases. The demographic and clinical characteristics of the participants are shown in Table 1.

Both 44 sequential sera from 11 HCC patients, who had a history of chronic hepatitis or liver cirrhosis and then developed HCC, and 48 sera from NCs were obtained from the sera bank in the Cancer Autoimmunity Research Laboratory at the University of Texas, El Paso (UTEP). Serial sera were collected every 3 months from HCC patients before and after diagnosis of HCC. Each patient had at least one, and up to 4 serum samples available before being diagnosed with HCC. This study was approved by the Institutional Review Board of the respective institutions.

### 2.2. Focused Protein Microarray

A focused protein microarray containing 154 proteins encoded by 138 cancer driver genes was constructed as described previously [16,31]. The detailed procedure for the detection of multiple autoantibodies including autoantibody to GNAS in HCC and NC sera was described in our recent study [16,31]. The level of autoantibody to GNAS was measured by the signal-to-noise ratio (SNR).

### 2.3. Enzyme-Linked Immunosorbent Assay (ELISA)

The GNAS recombinant protein used in the ELISA was purchased from AVIVA System Biology (San Diego, CA, USA) and Cloud-Clone Corporation (Wuhan, China). The coating concentration was 0.5 μg/mL. Sera were diluted at 1:100. A detailed procedure was described in our previous studies [16,31]. An ABTS color rendering system was used for the detection of autoantibody to GNAS in 44 human HCC successive sera and 48 NC sera; the details were seen in a previous study [15]. For every test, two blank controls without target recombinant protein and 8 fixed human serum samples were set up on each 96-well plate for the adjustment of background and the normalization of the OD value among different plates, respectively.

### 2.4. Statistical Analysis

Statistical analysis was carried out using Prism software (Version 6, GraphPad, La Jolla, CA, USA) and SPSS (Version 21.0, Chicago, IL, USA). Receiver operating characteristic (ROC) curves were generated and the area under the ROC curve (AUC) with sensitivity and specificity together was used to evaluate the diagnostic value of the autoantibody. Non-parametric tests (Mann–Whitney U test and Kruskal–Wallis H-test) were used for the significance analysis in SNR or OD values among two or multiple groups. The cutoff value was defined as the corresponding point of the largest Youden index, while the minimum specificity is 80% in both datasets. The Pearson Chi-square test was used to determine the significance of the frequency of the autoantibody among different groups in each dataset. *p* values less than 0.05 were considered to be significant.

## 3. Results

### 3.1. Study Design

**Figure 1:**
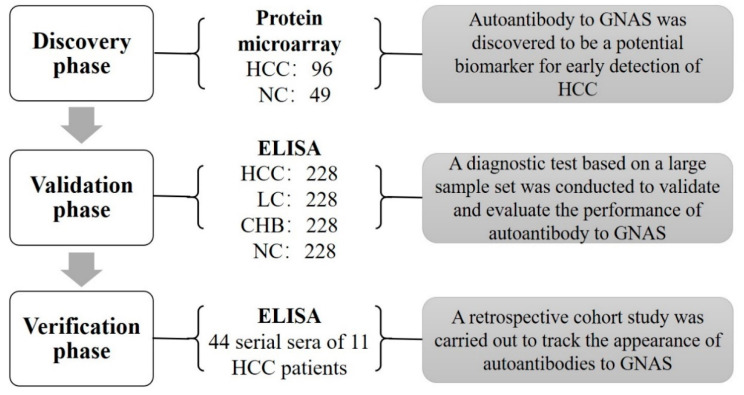
Flowchart of the study. A total of 145 subjects were first used to screen biomarkers in the discovery phase, autoantibody to GNAS was found to be a significant indicator for HCC. Subsequently, 912 participants in an independent set were included in the validation phase to validate and evaluate the performance of autoantibody to GNAS and finally, 44 serial serum samples of 11 HCC patients were used to track the appearance of autoantibody to GNAS. HCC: hepatocellular carcinoma; NC: normal control; LC: liver cirrhosis; CHB: chronic hepatitis B; ELISA: enzyme-linked immunosorbent assays.

As shown in Figure 1, there were three phases in this study: the discovery phase, validation phase, and verification phase. The first testing of sera from 96 HCC patients and 49 NCs in the discovery phase was performed on a focused protein microarray to explore the performance of autoantibody to GNAS across subgroups of HCC patients and normal controls. The following validation test, based on a large-scale serum sample set including 228 sera from HCC patients, 228 sera from LC patients, 228 sera from CHB patients, and 228 sera from NCs was conducted by ELISA to further detect and validate whether autoantibody to GNAS appeared with a trend in the patients at different stages. In the verification phase, 44 serial sera from 11 HCC patients were used to observe and evaluate the dynamic change of anti-GNAS autoantibody during the progression of chronic liver disease to HCC.

### 3.2. Performance of Autoantibody to GNAS in Sera from HCC Patients with Early and Late Stages in Discovery Phase

**Figure 2:**
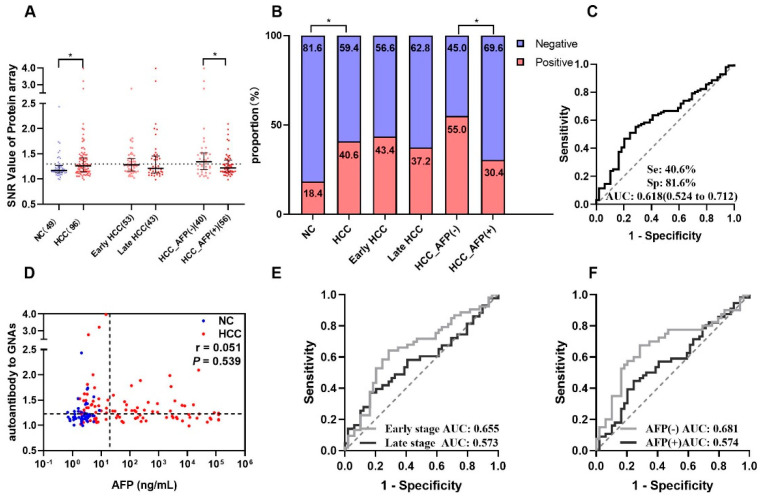
Autoantibody to GNAS was discovered by using a protein microarray. (**A**) The scatter plot of signal/noise ratio (SNR) to autoantibody to GNAS in different groups, the normal control group was shown in blue, HCC group and its subgroups were shown in red, while the cutoff value of 1.299 was determined by the maximum Yuden index with a specificity of 81.6%. (**B**) The distribution of positive rate and negative rate in different groups following the cutoff value of (**A**). The receiver operating characteristic curves (ROC) of autoantibody to GNAS for HCC patients, early-stage and late-stage HCC patients, AFP (−) and AFP (+) HCC patients are respectively demonstrated in (**C**,**E**,**F**). Correlation analysis was performed to explore the relationship of autoantibody to GNAS and AFP (**D**). HCC: hepatocellular carcinoma; NC: normal control; AFP: alpha-fetoprotein; Se: sensitivity; Sp: specificity; AUC: area under the receiver operating characteristic curve; 95% CI of AUC in brackets. The r in Figure 2D is the correlation coefficient. * *p* < 0.05. The two dotted lines in (**D**) are cutoff values of AFP (x = 20) and autoantibody to GNAS (y = 1.299), respectively.

The results from the focused protein microarray showed that autoantibody to GNAS in level and frequency was significantly higher in HCC patients than that in normal controls; it can distinguish 40.6% of HCC patients from normal controls with an AUC of 0.618 (Figure 2A–C). The stratification analysis indicated that autoantibody to GNAS in early-stage HCC patients (sensitivity of 43.4% and AUC of 0.655) presented better performance than that in late-stage HCC patients (sensitivity of 37.2% and AUC of 0.573) although the difference was not significant (Figure 2B,E). As shown in Figure 2B,F, its ability to diagnose AFP (−) HCC patients (sensitivity of 55.0% and AUC of 0.681) also seemed stronger than its ability to diagnose AFP (+) HCC patients (sensitivity of 30.4% and AUC of 0.574).

### 3.3. Validation in a Large-Scale Sample Set

**Figure 3:**
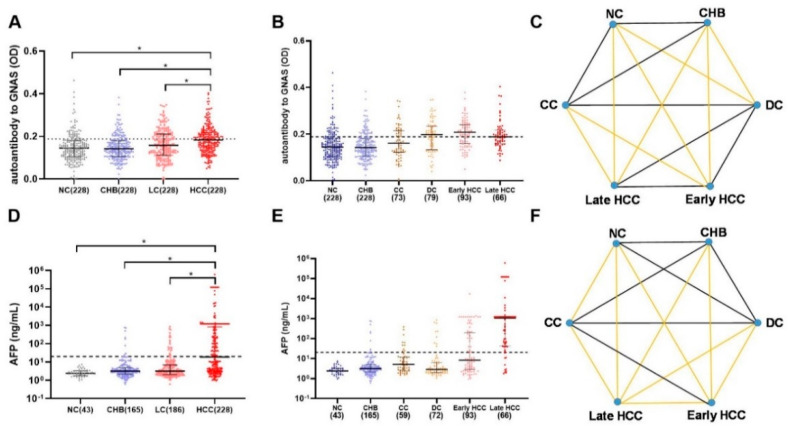
The scatter plots of autoantibody to GNAS and AFP in different groups of the development of HCC in the validation phase (**A**,**B**,**D**,**E**), the dots in different colors representing the different groups of the research objects. The statistical differences between each two groups in (**B**,**E**) are showed in (**C**,**F**). If the lines in (**C**,**F**) are yellow, the difference between groups of its two ends was considered to be significant (*p* < 0.05, * *p* < 0.05 in **A**,**D**). HCC: hepatocellular carcinoma; NC: normal control; LC: liver cirrhosis; CHB: chronic hepatitis B; CC: compensated liver cirrhosis; DC: decompensated liver cirrhosis; AFP: alpha-fetoprotein. The dotted line (y = 0.188) in Figure 3A,B shows the cut-off value of autoantibody to GNAS, while the cutoff value of AFP in Figure 3D,E was 20 ng/mL.

**Figure 4:**
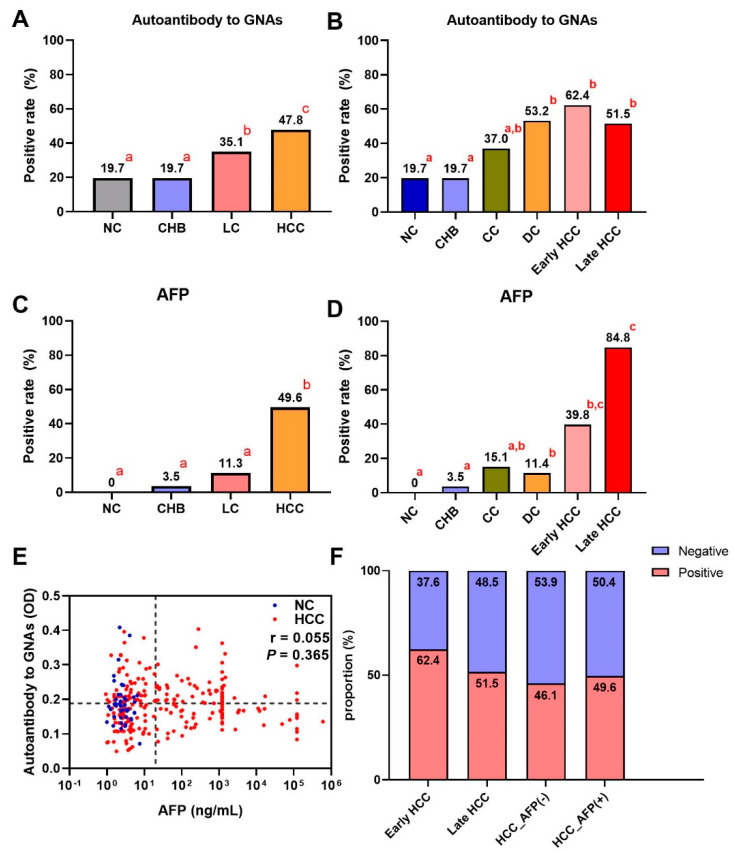
The bar charts of positive rates of autoantibody to GNAS and AFP in different groups of the development of HCC (**A**–**D**) in the validation phase. If the letters in the upper right corner (a, b, c) between two bars are totally different, the difference between the two groups is considered to be significant (*p* < 0.05). (**E**) The relationship of AFP and autoantibody to GNAS, and (**F**) the distribution of positive rate and negative rate in subgroups of HCC. HCC: hepatocellular carcinoma; NC: normal control; LC: liver cirrhosis; CHB: chronic hepatitis B; CC: compensated liver cirrhosis; DC: decompensated liver cirrhosis; AFP: alpha-fetoprotein. The r in (**E**) is the correlation coefficient.

A total of 912 participants were recruited into a large-scale sample set including 228 HCC patients, 228 LC patients, 228 CHB patients, and 228 NCs. As shown in Figure 3A, autoantibody to GNAS showed a gradual increase trend during the transition from chronic liver disease to HCC, but only the difference between HCC and each of the other three groups was significant. Figure 4A exhibited that autoantibody to GNAS was detected in 109 of 228 (47.8%) of HCC patients, which was a significantly higher frequency than in LC patients (80 of 228, 35.1%), CHB patients (45 of 228, 19.7%), and normal controls (45 of 228, 19.7%) with the significant difference between LC patients and CHB patients or NCs. The application of autoantibody to GNAS among different groups was also analyzed, and multiple parameters such as AUC, sensitivity (Se), specificity (Sp), positive predictive value (PPV), negative predictive value (NPV), false negative rate (FNR), and false positive rate (FPR) were calculated to reflect the diagnostic value of the autoantibody. As shown in Figure S1 and Table S1, when autoantibody to GNAS was used in distinguishing HCC from normal controls or CHB patients, the performance was the best with an AUC of 0.676 or 0.677 and PPV of 70.8%.

In further analysis, based on the HCC staging of 159 HCC patients, 93 HCC patients were in early-stage and 66 patients in late-stage. As shown in Figure 3B,C as well as Figure 4B,F, the data indicated that the level and positive rate (62.4%, 58/93) of autoantibody to GNAS in early-stage HCC patients was still higher than that (51.5%, 34/66) in late-stage HCC patients even if the difference was not significant, which was in line with the results from the discovery phase. Moreover, the ability of the autoantibody to distinguish early HCC patients from different control groups was slightly stronger than its ability to distinguish late HCC patients from different control groups (Table S1 and Figure S1). Based on the results of the liver function test and clinical manifestations, 152 LC patients with complete clinical information were defined as patients with compensated cirrhosis (CC, *n* = 73) and patients with decompensated cirrhosis (DC, *n* = 79). The positive rate (53.2%, 42/79) of autoantibody to GNAS in DC patients reached the same significance level (62.4% and 61.5%) in early- and late-stage HCC patients (Figure 4B).

After dividing patients with HCC and LC into subgroups, increasing levels of autoantibody to GNAS (Figure 3B,C) and frequencies of 19.7%, 19.7%, 37.0%, 53.2%, 62.4%, and 51.5% in NCs and patients with CHB, CC, DC, early-stage HCC, and late-stage HCC, respectively (Figure 4B) were observed. What attracts our attention is that autoantibody to GNAS in level and frequency started to increase in CC patients with a jump in DC patients and reached a peak in early HCC patients. The frequency of the autoantibody in DC patients was significantly higher than that in normal controls as well as CHB patients. As such, taking the results of the significance tests in Figure 4A,B together, the patients with CC and DC in our study were identified as high-risk individuals of HCC, and the patients with DC as super high-risk individuals of HCC or pre-HCC patients.

### 3.4. Dynamic Change of Anti-GNAS Autoantibody in Serial Sera from 11 HCC Patients for Follow-Up Evaluation

**Figure 5:**
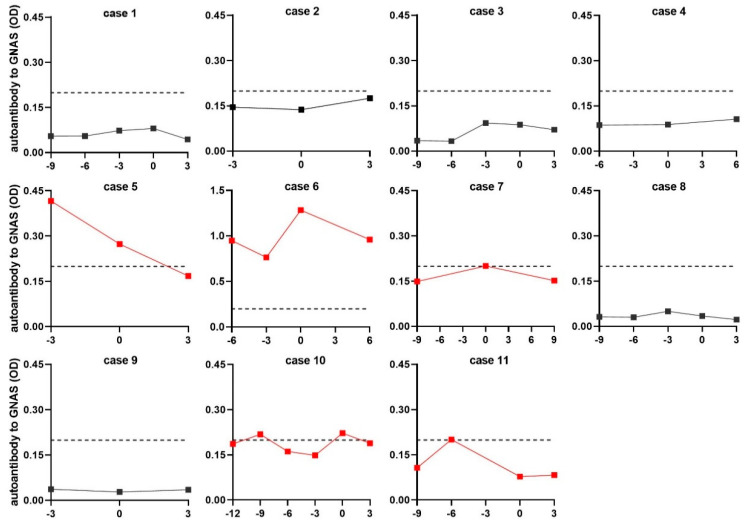
The level of autoantibody to GNAS in serial sera. The red lines refer to the serial sera with positive result from HCC patients before or at diagnosis of HCC. The vertical axis represents the OD value of autoantibody to GNAS, the horizontal axis represents the time frame (months) of serum sample collection. The “0” on the horizontal axis refers to the time the patient was diagnosed with HCC. Points to the left of “0” are the sera collected before diagnosis, points to the right of “0” are the sera collected after diagnosis. Each dot means each time node of serum sample collection. The dotted line parallel to the horizontal axis was cut-off lines (y = 0.199), which was determined by the 90th percentile of normal control.

An acceptable viewpoint is that if an autoantibody appears in patients at a precancerous stage or early HCC stage, it can most likely be considered as an early detection biomarker for HCC. To further confirm the value of autoantibody to GNAS in early diagnosis of HCC, another independent dataset comprising 48 NC sera and 44 serial sera from 11 HCC patients, who were tracked more than one year before and after diagnosis of HCC, was applied for follow-up evaluation. All 11 HCC patients had a history of chronic hepatitis or cirrhosis. One to four serum samples were collected from each patient every three months before diagnosis. The testing result and variation of autoantibody to GNAS in serial sera from each patient were depicted in line graphs (Figure 5). When the cut-off value was determined to be the 90th percentile of normal controls, 5 out of 11 patients (45.5%) showed positive results with autoantibody to GNAS at some time points prior to, or at diagnosis of HCC. In two of five positive patients developing HCC from pre-existing chronic liver disease, a high level of anti-GNAS autoantibody already existed before diagnosis with the peak at the point of diagnosis (Case 6, Case 7, and Case 10). In two of five positive patients, the elevated autoantibody to GNAS with a peak even appeared in patients 3 or 6 months ahead of the diagnosis of HCC (Case 5, Case 11). These results further confirmed more than 40% of HCC patients presented elevated anti-GNAS autoantibody levels in sera at, or prior to the diagnosis of HCC, suggesting that GNAS might be an early marker of transformation and the corresponding autoantibody may be a potential biomarker for early detection of HCC.

### 3.5. Complementary Effects of Anti-GNAS Autoantibody on AFP in HCC Detection

AFP testing result was available for most HCC patients in our study. As shown in Table 1, Figure 2B and Figure 4A,C, the sensitivities of AFP and autoantibody to GNAS in HCC patients were 58.3% and 40.6% in the discovery phase, and 49.6% and 47.8% in the validation phase, respectively. The correlation analysis of AFP and autoantibody to GNAS was performed and it was found that there was no correlation between autoantibody to GNAS and AFP in both the discovery (*r* = 0.051, *p* = 0.539) and validation phases (*r* = 0.055, *p* = 0.365) (Figure 2D and Figure 4E). When AFP and autoantibody to GNAS were combined to classify HCC patients and NCs, the sensitivities were elevated in both the discovery phase and validation phase (Table S2). The validation phase included a larger amount of HCC sera samples (*n* = 228) than the discovery phase (*n* = 96), so we focused on and used the data from the validation phase for further analysis. With an AFP value of 20 ng/mL as the cut-off, 228 HCC patients in the validation phase were divided into an AFP positive (AFP (+)) group and AFP negative (AFP (−)) group. The results of the further analysis indicated that autoantibody to GNAS can not only distinguish 46.1% of AFP (−) HCC patients from normal controls but also has a stronger ability to distinguish early HCC patients (62.4%) from normal controls than its ability to distinguish late HCC (51.5%) from normal controls (Figure 4B,F), which can supplement AFP in the diagnosis of HCC. This notion was also supported by the following finding: As seen in Figure 3A–F and Figure 4A–D, in patients at different stages of transition from chronic liver diseases to HCC, the autoantibody to GNAS in level and frequency gradually increased from CC through DC to HCC and reached the peak at the early HCC stage. Conversely, the AFP level just started to increase at the early HCC stage and reached a peak at the late stage.

## 4. Discussion

Hepatic carcinogenesis is a slow and long process involving multiple factors and multiple steps. Chronic hepatitis B virus (HBV) infection is a major risk factor for cirrhosis and hepatocellular carcinoma (HCC), accounting for over 50% of total HCC cases worldwide [32,33]. Chronic inflammation impacts every single step of tumorigenesis in the liver, from initiation to tumor promotion, all the way to metastatic progression through a series of mechanisms and processes, such as immune suppression of the microenvironment, chronic necroinflammation, repair and regeneration, induction of liver fibrosis, and subsequent cirrhosis [34,35,36]. From the perspective of population epidemiology and the etiology of HCC, most HCCs develop through a progressive pathway from precancerous lesions to cancerous lesions in the cirrhotic liver [33,37], and CHB and LC have been recognized as high-risk factors for HCC [38,39]. Based on Japanese liver cancer screening guidelines, people with LC are even more defined as the ultra-high-risk population for HCC [40]. However, from a pathological point of view, early HCC is small, indistinctly nodular, and highly differentiated, which results in the difficulty in distinguishing small, well-differentiated HCC masses from high-grade dysplastic nodules. Thus, the early HCC and high-grade dysplastic nodules may exist at the same time and some LC patients actually have early-stage HCC [41].

*GNAS*, as one of the most common mutated genes, has been shown to be closely related to cancers. Its activating mutations were associated with uncontrolled intracellular cAMP accumulation leading to cellular proliferation and tumor formation through the stimulation of different signaling pathways [42,43]. It was found in Nault’s study that the activation of signaling pathways caused by *GNAS* activating mutations and the inflammatory effect of STAT3 activation may have synergistic effects in hepatocellular carcinogenesis [44]. Ding’s study in HCC cell lines also demonstrated that GNAS played a tumor-promoting role in inflammation-related HCC progression [27]. During the process of tumorigenesis, GNAS changes in quantity and quality, which may trigger an immune response leading to autoantibody production in cancer patients [45]. The occurrence and elevation of autoantibody to GNAS in HCC patients was explored in our recent study [17].

In the current study, we found that autoantibody to GNAS in level and frequency was significantly higher in HCC patients than that in normal controls. Higher autoantibody response to GNAS was also found in early-stage HCC patients with early-stage compared to late-stage HCC patients in both the discovery and validation phases. The autoantibody to GNAS can distinguish more HCC patients with early-stage (43.4% and 62.4%) than HCC patients with late-stage (32.6% and 51.5%) in discovery and validation phases, respectively. These results suggested that a stronger immune response to GNAS occurs in the early stage of HCC patients and cannot be enhanced with the progression of HCC after the formation of malignant tumors. Evidence in Pardoll’s and O’Donnell’s reviews indicated that specific immune activation systems operate at the early stages of tumorigenesis. As the immune tolerance has not been established in this stage, the body has a stronger immune response to TAAs, while established tumors primarily induce immune tolerance [46,47]. This possible mechanism explained that autoantibody to GNAS elevated in early-stage HCC patients without further increase as the cancer progressed. It is precisely because autoantibody to GNAS appears in the sera from early HCC patients that it is possible to use it as an indicator for the early detection of HCC.

As mentioned above, most human HCCs are accompanied by chronic diseases such as chronic hepatitis and liver cirrhosis. To detect and validate the autoantibody response to GNAS in patients at different stages of transition from chronic liver disease to HCC, a large-scale sample set (*n* = 912) was established to further evaluate the possibility of autoantibody to GNAS as a serum biomarker for early detection of HCC. The results indicated that the frequency of autoantibody to GNAS gradually increased with the transition from CHB, LC to HCC. When LC and HCC patients were subdivided into different groups by stage, the positive rates of the autoantibody response to GNAS were 19.7%, 19.7%, 37%, 53.2%, 62.4%, and 51.5% in NCs and patients with CHB, CC, DC, early HCC, and late HCC, respectively, showing a close association of the autoantibody with the progression of chronic liver disease to HCC. The significant increase of autoantibody to GNAS in pre-HCC and early HCC patients may be due to the occurrence of GNAS protein change in quality and quantity which might be recognized as heterologous antigens by the immune system to trigger a humoral immune response for producing the corresponding autoantibody. This also implied that autoantibody to GNAS could potentially be used as a serum biomarker for the early detection of HCC. Based on Kojiro’s discovery [41], it was speculated that the increased autoantibody to GNAS in patients with liver cirrhosis might be due to the undetectable early HCC patients occupying a certain proportion. Another opinion from Rizzo’s viewpoint [37] is that most changes found in HCC occur in low-grade dysplastic nodules, which is the earliest known stage of hepatocarcinogenesis. As for the undetectable positive autoantibody response to GNAS in patients with CHB, it might be related to immunosuppression induced by viruses in the inflammation stage [32]. This finding is similar to the previous research results on small samples which exhibited the frequency of an eight-TAAb panel in HCC (59.8%), LC (30%), CH (20%), and NC (12.2%) [48].

The above-mentioned large sample testing was a case-control study performed on patients at different stages of liver diseases ranging from chronic liver diseases to HCC. If there was a series of sera from each of the same group of patients with transition from chronic liver diseases to HCC available for testing, the results obtained from the serial serum samples would be more credible. In our study, another validation cohort including 48 NC sera and 44 follow-up serum samples from 11 HCC patients addressed and confirmed this hypothesis. It was found that 5 out of 11 (45.5%) HCC patients had positive detection with autoantibody to GNAS before or at diagnosis of HCC even though the level and change of anti-GNAS autoantibody varied in serial sera for each patient. In four of five HCC positive patients, the autoantibody to GNAS reached the peak at or before the diagnosis of HCC. The finding suggested that autoantibody response to GNAS might already be produced in the pre-HCC stage, and further confirmed that autoantibody to GNAS has more potential as an early diagnostic biomarker for HCC. In Meistere’s study [7], 18 gastric cancer patients were tracked for many years before diagnosis and it was found that autoantibody response against five TAAs was detected in serum samples taken a few years before the clinical diagnosis of these gastric cancer patients, which was similar to the finding from our current study.

AFP is the most commonly used serum biomarker for auxiliary diagnosis of HCC in the clinic, but the deficiency of low sensitivity becomes obvious when it is used to diagnose early HCC [4]. The addition of a panel of 10 TAAbs to AFP could significantly raise the sensitivity of HCC detection, and the positive rate of the combination of both was significantly associated with the increasing stage of HCC [49]. In the current study, an interesting finding was the difference in a trend change and the possibility of complementarity between autoantibody to GNAS and AFP. The sensitivity of autoantibody to GNAS was significantly associated with increasing stages of HCC development with no change from early-stage to late-stage HCC. However, the elevation of AFP level only occurred in HCC patients and was correlated with a more aggressive stage of HCC, which was consistent with the finding in Nishioka’s study [50]. Moreover, 62.4% of early-stage HCC patients and 46.1% of AFP (−) HCC patients showed positive detection with anti-GNAS autoantibody. Therefore, these results implied that the autoantibody to GNAS might have complementary effects on AFP in the detection of HCC.

## 5. Conclusions

In summary, 228 subjects in each of four groups in the validation phase were matched in age and gender, except CHB patients in age so that the results are more reliable and comparable. The results from the large-scale sample set demonstrated that the frequency of autoantibody to GNAS increased not only in patients with early HCC but also in patients with decompensated LC (pre-HCC patients). Follow-up testing in human serial sera from 11 patients who had developed HCC from chronic liver disease provided more sufficient evidence that autoantibody to GNAS might be considered as a serum biomarker for early detection of HCC. The autoantibody to GNAS can supplement the limitation of AFP. Limitations of the current study are that even though 44 successive sera from 11 HCC patients were available for clinical follow-up evaluation, the timeline of follow-up was less than 2 years before and after diagnosis of HCC, and the number of follow-up patients was not large enough. Thus, a long-term prospective study on high-risk individuals will be conducted in the future.

## Figures and Tables

**Table 1:** Characteristics of participants.

	Discovery Phase		Validation Phase			
	HCC	NC	HCC	NC	LC	CHB
N	96	49	228	228	228	228
Gender, *n* (%)						
Male	79 (82.3)	22 (44.9)	187 (82.0)	187 (82.0)	187 (82.0)	179 (78.5)
Female	17 (17.7)	27 (55.1)	41 (18.0)	41 (18.0)	41 (18.0)	49 (21.5)
Age, years						
Range	37–78	20–71	20–75	21–73	23–75	23–79
Mean ± SD	56.7 ± 9.3	40.1 ± 12.8	52.3 ± 10.7	51.4 ± 10.2	52.2 ± 10.5	45.2 ± 11.1
AFP, *n* (%)						
AFP (+)	56 (58.3)	0 (0)	113 (49.6)	0 (0)	21 (9.2)	8 (3.5)
AFP (−)	40 (41.7)	49 (100)	115 (50.4)	43 (18.9)	165 (72.4)	157 (68.9)
NA	0 (0)	0 (0)	0 (0)	185 (81.1)	42 (18.4)	61 (26.8)
HCC stage, *n* (%)						
Early-stage	53 (55.2)		93 (40.8)			
Late-stage	43 (44.8)		66 (28.9)			
NA	0 (0)		69 (30.3)			
LC stage, *n* (%)						
CC					73 (30.0)	
DC					79 (34.7)	
NA					76 (33.3)	

HCC: hepatocellular carcinoma; NC: normal control; LC: liver cirrhosis; CHB: chronic hepatitis B; CC: compensated cirrhosis; DC: decompensated cirrhosis; SD: standard deviation; AFP: alpha-fetoprotein; NA: not available; AFP (+): AFP ≥ 20 ng/mL, AFP (−): AFP < 20 ng/mL.

## Data Availability

Data are available on request to the corresponding author.

## Supplementary Materials

The following are available online at https://www.mdpi.com/article/10.3390/biomedicines10010097/s1, Figure S1: ROCs of autoantibody to GNAS in different cohorts in the validation phase. HCC: hepatocellular carcinoma; NC: normal control; LC: liver cirrhosis; CHB: chronic hepatitis B; AUC: area under the receiver operating characteristic curve; 95% CI of AUC in brackets; Se: sensitivity; Sp: specificity, Table S1: The diagnostic performance of autoantibody to GNAS in different cohorts of the validation phase. Table S2. The diagnostic performance of the combination autoantibody to GNAS with AFP in different phase when distinguish HCC from NC.
