# Supplementary material for: Autoantibody to GNAS in Early Detection of Hepatocellular Carcinoma: A Large-Scale Sample Study Combined with Verification in Serial Sera from HCC Patients
Source: Biomedicines. 2022 Jan 4;10(1):97. doi: 10.3390/biomedicines10010097 (PMC8773227; doi:10.3390/biomedicines10010097)
Supplement: Supplementary file 1 [file biomedicines-10-00097-s001.zip › biomedicines-1534387-supplementary.pdf]

Table S1. The diagnostic performance of autoantibody to GNAS in different cohorts of validation phase

Cohorts	AUC	*P*	Se (%)	Sp (%)	PPV (%)	NPV (%)	FNR (%)	FPR (%)
HCC vs NC	0.676	<0.0001	47.8	80.3	70.8	60.6	52.2	19.7
HCC vs LC	0.596	0.0004	47.8	64.9	57.7	55.4	52.2	35.1
HCC vs CHB	0.677	<0.0001	47.8	80.3	70.8	60.6	52.2	19.7
HCC vs (LC+CHB)	0.637	<0.0001	47.8	72.6	46.6	73.6	52.2	27.4
HCC vs (NC+LC+CHB)	0.650	<0.0001	47.8	75.1	39.1	81.2	52.2	24.9
Early HCC vs NC	0.757	<0.0001	62.4	80.3	56.3	83.9	37.6	19.7
Early HCC vs LC	0.678	<0.0001	62.4	64.9	42.0	80.9	37.6	35.1
Early HCC vs CHB	0.760	<0.0001	62.4	80.3	56.3	83.9	37.6	19.7
Early HCC vs (LC+CHB)	0.719	<0.0001	62.4	72.6	31.7	90.4	37.6	27.4
Early HCC vs (NC+LC+CHB)	0.732	<0.0001	62.4	75.1	25.4	93.6	37.6	24.9
Late HCC vs NC	0.738	<0.0001	51.5	80.3	43.0	85.1	48.5	19.7
Late HCC vs LC	0.642	0.0004	51.5	64.9	29.8	82.2	48.5	35.1
Late HCC vs CHB	0.742	<0.0001	51.5	80.3	43.0	85.1	48.5	19.7
Late HCC vs (LC+CHB)	0.692	<0.0001	51.5	72.6	21.4	91.2	48.5	27.4
Late HCC vs (NC+LC+CHB)	0.708	<0.0001	51.5	75.1	16.7	94.1	48.5	24.9

HCC: hepatocellular carcinoma, NC: normal control, LC: liver cirrhosis, CHB: chronic hepatitis B, AUC: area under the receiver operating characteristic curve, CI: confidence interval, Se: sensitivity, Sp: specificity, PPV: positive predictive value, NPV: negative predictive value, FNR: false negative rate, FPR: false positive rate.

Table S2. The diagnostic performance of the combination autoantibody to GNAS with AFP in different phase when distinguish HCC from NC.

Biomarkers	Sensitivity(%)	Specificity(%)
Discovery phase		
GNAS	40.6	81.6
AFP	58.3	100.0
GNAS or AFP	81.3	81.6
Validation phase		
GNAS	47.8	80.3
AFP	49.8	100.0
GNAS or AFP	72.8	80.3

AFP: alpha-fetoprotein; GNAS: autoantibody to GNAS. The cut off value of AFP and autoantibody to GNAS were 20 ng/mL and 0.188 (OD), respectively.

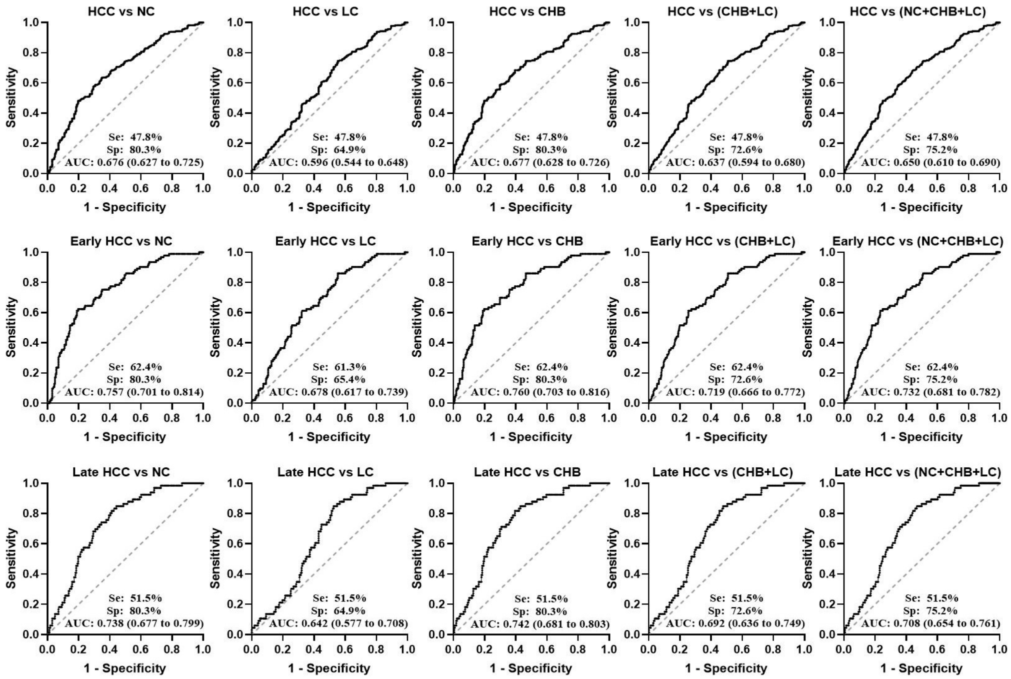

Figure S1. ROCs of autoantibody to GNAS in different cohorts in validation phase. HCC: hepatocellular carcinoma, NC: normal control, LC: liver cirrhosis, CHB: chronic hepatitis B, AUC: area under the receiver operating characteristic curve, 95% CI of AUC in brackets, Se: sensitivity, Sp: specificity.
